# Geospatial dataset for analyzing socio-economic regional divergence of European regions

**DOI:** 10.1016/j.dib.2018.07.027

**Published:** 2018-07-19

**Authors:** Andrey S. Mikhaylov, Anna A. Mikhaylova, Tatyana Yu. Kuznetsova

**Affiliations:** Immanuel Kant Baltic Federal University, 23016 Kaliningrad, Russian Federation

## Abstract

This data article presents macroeconomic data that can be used for comparative territorial studies. The data cover a sample of 413 regions (national administrative-territorial units corresponding to second level of a common classification of territorial units for statistics of the European Commission – NUTS 2 level region of the European Union, and comparable administrative-territorial units outside the EU) of 48 European countries, including Cyprus, Turkey, the European part of Russia, and two partially recognized states – the Republic of Kosovo and the Pridnestrovian Moldavian Republic. The statistical database covers a five-year period of 2010–2014. This dataset is created to enhance our understanding of the contemporary coastalization dynamics in Europe. Despite the fact that coastal regions of European countries exhibit an extensive level of development and remain attractive to human settlement, industry localization, and investment flows their contribution to the socio-economic development of Europe is unclear. The reported data cover a series of macroeconomic data on key indicators traditionally used in comparative analysis of regional development: average annual population, gross regional product (GRP) in purchasing power parity (PPP), labor productivity, population density and GRP (PPP) values per sq.km. Accounting for differences in geoeconomic position of the European regions enables to distinguish four subtypes of regions with a particular emphasis on the coastal area: coastal border, coastal other, coastal hinterland, and inland other. An additional focus is made on differentiating the performance indicators of regions depending on their border geo-economic position: border regions with a state border over land, lake or river surface, and midland regions – other non-border regions. This data is to be used as a comparative benchmark for the coastal border subgroup of regions against the totality of border and midland regions.

## Specifications Table

**Table:** 

Subject area	Geography
More specific subject area	Human geography
Type of data	Figures, tables and Excel files
How data was acquired	Data are acquired from the International Monetary Fund (IMF), the World Bank Open Data, the International Bank for Reconstruction and Development (IBRD), the United Nations Statistics Division (UNSD), the Trading Economics portal, the Statistical Office of the European Union (Eurostat), and national statistical offices and authorities of each sample country: the National Bureau of Statistics of the Republic of Moldova, the Turkish Statistical Institute, the Economic Development Ministry of the Pridnestrovian Moldavian Republic, the Pridnestrovian Republican Bank, Monaco Statistics, the Office of Economic Planning, Data Processing and Statistics of the Republic of San Marino, the Institute of Statistics of the Republic of Albania, the Republika Srpska Institute of Statistics, the Kosovo Agency of Statistics, the National Statistical Committee of the Republic of Belarus, the Agency for statistics of Bosnia and Herzegovina, the Statistical Office of the Republic of Serbia, the Federal Service of State Statistics of the Russian Federation, the State Statistics Service of Ukraine, the Statistical Office of Montenegro, the Federal Statistical Office of Switzerland, the Government of Andorra official website, the Vatican City State
Data format	Aggregated, processed
Experimental factors	The sample was extracted by merging information from Eurostat, national statistical offices and authorities of the countries studied, the World Bank, IBRD, UNSD, IMF, and the Trading Economics portal. Sample processing involved converting the raw data collected from the various sources into a comparable form; data extrapolation to periods where data were not available; aggregation by types of regions; conversion of raw data into calculated indicators, growth rates and coefficients; ranking of regions.
Experimental features	The data presented cover a series of macroeconomic data on the most important indicators used in socio-economic studies when conducting a comparative analysis of the level of territorial development.
Data source location	Albania, Andorra, Austria, Belarus, Belgium, Bosnia and Herzegovina, Bulgaria, Croatia, Cyprus, Czech Republic, Denmark, Estonia, Finland, France, Germany, Great Britain, Greece, Hungary, Iceland, Ireland, Italy, Kosovo, Latvia, Liechtenstein, Lithuania, Luxembourg, Macedonia, Malta, Moldova, Monaco, Montenegro, Netherlands, Norway, Poland, Portugal, Romania, Pridnestrovie, Russia (European part), San Marino, Serbia, Slovakia, Slovenia, Spain, Sweden, Switzerland, Turkey, Ukraine, Vatican
Data accessibility	Data are available within this article

## Value of the data

• Studies on coastalization generally confirm the asymmetrical development of territories with gravitation towards marine and ocean coasts [1], [2], [3], [4], [5], [6], [7]. To some extend these results are predetermined by the research scope featuring islands and marine-focused economies, or a limited classification of territories – coastal and non-coastal, etc. The data presented responds to such research limitations by providing macroeconomic data across Europe for a broad classification of regions. Dataset enables to differentiate development patterns of coastal and adjacent regions (coastal hinterland), border regions, coastal borderland, and midland (inland) territories (regional typology is acquired from [8]).
• The dataset covers the entire territory of Europe, including countries that are not part of the European Union (EU). Difficulties in collecting and harmonizing the data of national statistical offices limit most available research to the EU or national level. The data provided enables to conduct comparative studies on regional socio-economic development across Europe, including the European part of Russia. Of particular value would be research on regional divergence at macro-regional level (e.g. Baltic region, Mediterranean region, Baltic-Black Sea region, etc.).
• This dataset may have important policy implications. The identifiable socio-economic development trajectories of regions over the five-year period my reveal distinct patterns in the development of regions of different types (e.g. the interrelation between coastal regions and the adjacent territories of coastal hinterland). Correlations may be found between the certain policy instruments implemented and the change in macroeconomic indicators. The data may be useful in developing socio-economic typologies of regions and assessing the differences in the territorial development of individual European countries.

## 1 Data

The data cover a sample of 413 regions (the level of NUTS 2 and comparable administrative-territorial units) of 48 European countries. Dataset spans over the period 2010–2014. The data is grouped according to the types of regions allocated on the basis of their geo-economic position (Table 1). The determining factor for assigning a region to a particular subgroup within the first group is its location relative to marine and ocean coasts (coastal geo-economic position): 1. – coastal; 1.1 – coastal border; 1.2 – coastal other; 2. – inland; 2.1 – coastal hinterland; 2.2 – inland other. Second group focuses on the borderland geo-economic position featuring two subgroups of regions: 1. – border; 2. – midland. The two groups are designed to be used complementary. Second group is designed to obtain a comparative benchmark for the coastal border subgroup of regions against the totality of border and midland regions.

**Table 1:** Dynamics of the main socio-economic indicators of regional development, 2010–2014.

**Indicator**	**Year**	**Unit**	**Total**	**Group I**						**Group II**	
				**1.**	1.1.	1.2.	**2.**	2.1.	2.2.	**1.**	**2.**
**Average annual population, mln people**	**2010**	total	**769.8**	**322.2**	142.0	180.2	**447.6**	180.1	267.4	**382.9**	**386.9**
		per region	**1.1**	**1.0**	1.3	0.9	**1.2**	1.5	1.1	**1.2**	**0.9**
	**2011**	total	**770.4**	**323.4**	142.0	181.4	**447.0**	180.2	266.8	**382.2**	**388.2**
		per region	**1.1**	**1.0**	1.3	0.9	**1.2**	1.5	1.1	**1.2**	**0.9**
	**2012**	total	**771.8**	**324.4**	142.1	182.3	**447.4**	180.4	267.1	**382.2**	**389.7**
		per region	**1.1**	**1.0**	1.3	0.9	**1.2**	1.5	1.1	**1.2**	**0.9**
	**2013**	total	**774.4**	**325.8**	142.2	183.5	**448.6**	181.1	267.5	**382.9**	**391.5**
		per region	**1.1**	**1.0**	1.3	0.9	**1.2**	1.5	1.1	**1.2**	**0.9**
	**2014**	total	**777.1**	**327.3**	142.4	184.9	**449.8**	181.8	268.0	**383.6**	**393.5**
		per region	**1.1**	**1.0**	1.3	0.9	**1.2**	1.5	1.1	**1.2**	**1.0**
**GRP in PPP, billion Euro**	**2010**	total	**15,932.0**	**6984.8**	2648.8	4336.0	**8947.2**	3957.6	4989.5	**6914.3**	**9017.7**
		per region	**22.8**	**22.0**	23.4	21.5	**24.0**	32.4	20.9	**21.9**	**21.8**
	**2011**	total	**16,399.9**	**7136.0**	2706.9	4429.0	**9264.0**	4086.7	5177.3	**7153.0**	**9246.9**
		per region	**23.5**	**22.4**	23.9	22.0	**24.9**	33.5	21.7	**22.6**	**22.4**
	**2012**	total	**17,078.3**	**7406.6**	2808.2	4598.5	**9671.7**	4208.0	5463.7	**7376.1**	**9702.2**
		per region	**24.4**	**23.3**	24.8	22.8	**26.0**	34.4	22.9	**23.3**	**23.5**
	**2013**	total	**17,234.9**	**7471.1**	2826.9	4644.2	**9763.8**	4249.0	5514.8	**7427.9**	**9807.0**
		per region	**24.7**	**23.5**	25.0	23.1	**26.2**	34.8	23.2	**23.5**	**23.8**
	**2014**	total	**17,740.9**	**7665.8**	2874.0	4791.8	**10075.1**	4368.9	5706.2	**7621.4**	**10119.5**
		per region	**25.4**	**24.1**	25.4	23.8	**27.1**	35.8	24.0	**24.1**	**24.5**
**Labor productivity, thousand euro in PPP per person**	**2010**	per region	**20.7**	**21.7**	18.7	24.1	**20.0**	22.0	18.7	**18.1**	**23.3**
	**2011**		**21.3**	**22.1**	19.1	24.4	**20.7**	22.7	19.4	**18.7**	**23.8**
	**2012**		**22.1**	**22.8**	19.8	25.2	**21.6**	23.3	20.5	**19.3**	**24.9**
	**2013**		**22.3**	**22.9**	19.9	25.3	**21.8**	23.5	20.6	**19.4**	**25.1**
	**2014**		**22.8**	**23.4**	20.2	25.9	**22.4**	24.0	21.3	**19.9**	**25.7**

Note: Group I regions: 1. – coastal; 1.1 – coastal border; 1.2 – coastal other; 2. – inland; 2.1 – coastal hinterland; 2.2 – inland other; Group II regions: 1. – border; 2. – midland.

The ‘per region’ indicator was calculated via multiplication of the median area of the regions of a particular subtype and the average weighted population density divided by GRP in PPP of the given subtype of regions

The choice of average annual population and GRP (PPP) data as the basic indicators for analysis is due, firstly, to their universality (they are taken into account in the statistical bases of all European countries or may be alternatively obtained from calculating the available statistical data); secondly, they reflect the level of regional socio-economic development, and in relative terms (per sq. km or per person) demonstrate the concentration of resources in a certain region, which is necessary to assess coastalization or regional divergence in general.

**Fig. 1:**
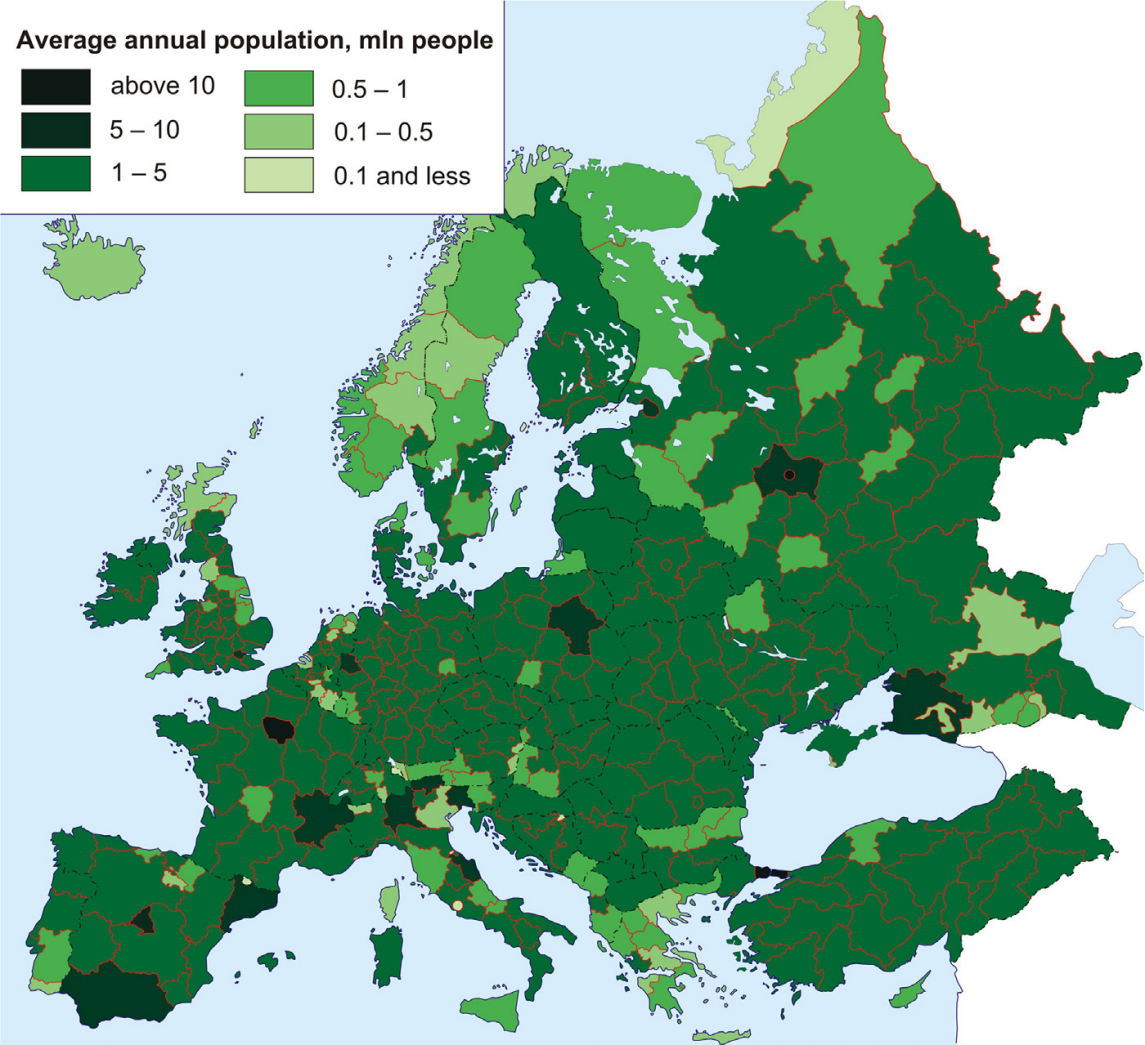
Average annual population, mln people.

**Fig. 2:**
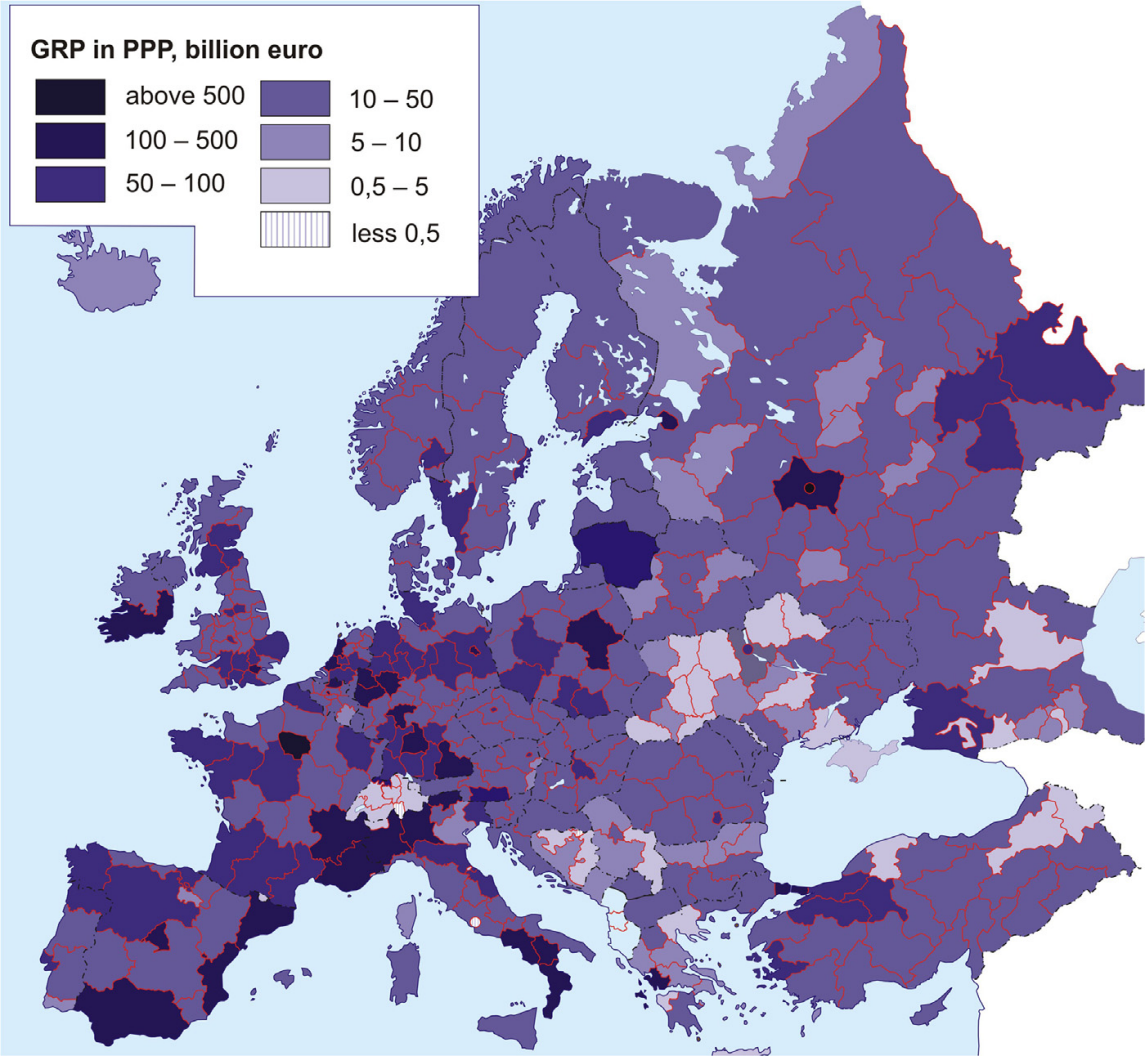
Gross Regional Product based on purchasing power parity, billion Euro.

Figs. 1 and 2 serve as clear representation of data on average annual population and GRP (PPP) in all regions of Europe – the average value for period 2010–2014. Raw data for a series of maps are available in Excel spreadsheets with a separate table for each map.

The Supplementary data provide the developed typology of European regions with an indication of the nomenclature of each region and country, as well as information on the size of territory (Supplement 1a–d and 2a,b). Individual Excel tables present a series of aggregated macroeconomic data on the types of European regions studied.

## 2 Experimental design, materials and methods

The data cover a sample of 413 regions of 48 European countries, including Cyprus, Turkey, the European part of Russia, and two partially recognized states – the Republic of Kosovo and the Pridnestrovian Moldavian Republic. Individual account for Kosovo and Pridnestrovie is required to obtain accurate statistical data (not estimates) and to consider their region types as de facto independent territorial socio-economic systems. The statistical data for the European territory of the Russian Federation includes regions of the Northwestern, Central, Volga, Southern, North Caucasus, and from 2014 the Crimean Federal Districts. Regions are defined as national administrative-territorial units corresponding to second level of a common classification of territorial units for statistics of the European Commission (2015) – NUTS 2 level region of the European Union, and comparable administrative-territorial units outside the EU. For 17 European states this classification corresponds to the total territory of the country (Andorra, Cyprus, Estonia, Iceland, Kosovo, Latvia, Liechtenstein, Lithuania, Luxembourg, Macedonia, Malta, Moldavia, Monaco, Montenegro, Pridnestrovie, San Marino, and Vatican). The NUTS 2 level of assessment represents holistic socio-economic systems, featuring a high degree of economic and institutional self-sufficiency. The classification corresponds to official statistics available for the overwhelming majority of countries under study, which provides the comparability of results and a possibility of building a complete series of statistical data by regions.

Regional sampling is made with following demarcation assumptions:

– all regions with marine coast are referred to as coastal regions regardless of the length of the shoreline (e.g. Federation of Bosnia and Herzegovina; Warmian-Masurian region (voivodeship) of the Republic of Poland for having access to the Vistula Lagoon and the Baltic Sea through the Kaliningrad Lagoon);
– all islands (e.g. Balearic) and island states (e.g. Cyprus) are referred to as ‘coastal other’ region subtype;
– Picardy region of France, the Kherson region of Ukraine and the Pomeranian region of Poland are accounted for as ‘coastal border’ regions;
– islands of France (Guadeloupe, Martinique, La Réunion, Mayotte), Portugal (Madeira, Azores), and Canary Islands of Spain are excluded from the study because of their considerable distance from mainland Europe;
– micro-enclaves, such as Jungholz (Austria), Baarle-Hertog (Belgium), Büsingen am Hochreihn (Germany), Livia (Spain), Campione d׳Italia (Italy), Baarle-Nassau (Netherlands), Dubrovnik (Croatia), Medvezhye-Sankovo (Russia) are not considered separately, while overseas enclaves of Spain in Africa (Ceuta, Melilla) and France in South America (Guyane) are excluded from the study;
– in case of the ‘enclave-like’ position of the region in the ‘coastal hinterland’, the region is equated to this subtype (e.g. Brussels-Capital Region, Berlin, Republic of Adygea);
– Greater London sub-regions are considered as a single region (Inner London – West, Inner London – East, Outer London – East and North East, Outer London – South, Outer London – West and North West) and assigned to ‘coastal other’ subtype of regions;
– regions with the port cities of Germany (Hamburg, Bremen) are considered as coastal.

The statistical database covers a five-year period of 2010–2014. The macroeconomic data is collected from several reliable sources, such as the Eurostat, national statistical offices, the World Bank, the IMF. When creating the database the comparability of the indicators’ measurement units was ensures (i.e. GRP (PPP) of all countries is quoted in Euro). For some regions, individual indicator values were either unavailable or inaccessible, so data extrapolation and interpolation techniques were applied to build complete data series. This is done in the following ways: if the value of the indicator for the region was known only for one year from the period under consideration, its value was taken as a constant and extrapolated for the entire period of 2010–2014; if the value of the indicator for the following year was omitted, it was replaced by data for the previous year; if the value of the indicator for the first analyzed year was not available, then it was replaced by data for the following year. Table 2 provides the variable definitions for the macroeconomic series.

**Table 2:** Variable definitions.

Variable definition	Data frequency	Source of data
*Basic indicators of regional socio-economic development level*		
Regional average annual population (in the absence of data calculated as arithmetic average of population figures as of January 1 of current year and as of January 1 of the following year)	Annually Data transposition is made for the regions of Slovenia, where the 2014 data is extrapolated for 2010–2013, and Albania, where the missing data for 2014 is replaced by the data for 2013	Austria, Belgium, Bulgaria, Croatia, Cyprus, Czech Republic, Denmark, Estonia, Finland, France, Germany, Great Britain, Greece, Hungary, Ireland, Italy, Latvia, Lithuania, Luxembourg, Macedonia, Malta, Netherlands, Norway, Poland, Portugal, Romania, Slovakia, Slovenia, Spain, Sweden: Eurostat Iceland: UNSD Liechtenstein: Trading Economics portal Montenegro: Statistical Office of Montenegro Switzerland: Federal Statistical Office Andorra: Government of Andorra official website, World Bank Open Data, IMF; Moldova: National Bureau of Statistics of the Republic of Moldova; Turkey: Turkish Statistical Institute; Pridnestrovie: the Economic Development Ministry, Pridnestrovian Republican Bank; Monaco: Monaco Statistics; Albania: the Institute of Statistics of the Republic of Albania; San Marino: the Office of Economic Planning, Data Processing and Statistics of the Republic of San Marino, United Nations Statistics Division; Serbia: Statistical Office of the Republic of Serbia; Kosovo: Kosovo Agency of Statistics; Belarus: National Statistical Committee of the Republic of Belarus; Bosnia and Herzegovina: Agency for statistics of Bosnia and Herzegovina; Serbia: Statistical Office of the Republic of Serbia; Russia: Federal Service of State Statistics of the Russian Federation; Ukraine: State Statistics Service of Ukraine; Vatican: Vatican City State, World Bank Open Data, IMF
Gross regional product (GRP) in purchasing power parity (PPP) in Euro	Annually Data transposition is made for the regions of Norway: the missing data of 2010 is replaced by that of 2011, and for 2014 – by that of 2013; for Albania, the – 2014 data is replaced by that of 2013; for Liechtenstein – 2013 by 2014; for Serbia – 2010, 2011, 2012 by 2013; and for Monaco – 2012, 2013, 2014 by 2011.	
Area of the region	Fixed indicator value as of 2014	

*Indicator of regional economic efficiency*		
Regional labor productivity, thousand euro in PPP per person	Annually	Ratio of GRP (PPP) to average annual population

*Indicators of regional socio-economic development dynamics*		
Average annual population growth rate	Annually	Ratio of average annual population in 2014 relative to 2010
GRP (PPP) growth rate	Annually	Ratio of GRP (PPP) in 2014 relative to 2010
Labor productivity growth rate	Annually	Ratio of labor productivity in 2014 relative to 2010

*Indicators of regional development patterns by region type distribution*		
Average annual average population by region type, thousand people	Annually	Ratio of the total average annual population by region type to the number of regions of this type
Average GRP (PPP) by region type, billion Euro	Annually	Ratio of the total GRP (PPP) by region type to the number of regions of this type
Average area by region type, thousand sq.km	Fixed indicator value as of 2014	Ratio of the total area by region type to the number of regions of this type

*Indicators of regional concentration of resources*		
Average concentration of the average annual population by region type, people per 1000 sq.km	Annually	Ratio of the total average annual population by region type to the total area of the regions of this type
Average concentration of GRP (PPP) by region type, million Euro per 1000 sq.km	Annually	Ratio of total GRP (PPP) by region type to the total area of the regions of this type

Aggregation of the initial data was performed in the context of the proposed classification of regions to obtain the values of average annual population and GRP (PPP) indicators by the types and subtypes of regions. At the next stage, the data was converted into calculated indicators, growth rates, coefficients. The ranking of regions was carried out.

Firstly, there are basic indicators of regional socio-economic development level and regional economic efficiency. These indicators are used to assess manifestation of coastalization in Europe, as well as for segregation of European regions featuring different geo-economic position in terms of socio-economic development.

Secondly, there are indicators of regional socio-economic development dynamics. They are used to assess regional development vector, namely, the growth or decline of average annual population, GRP, labor productivity.

Thirdly, there are indicators of the typological distribution of regions. They are used to assess distribution patterns of population and GRP (PPP) between the identified types of regions: Group I regions: 1. – coastal; 1.1 – coastal border; 1.2 – coastal other; 2. – inland; 2.1 – coastal hinterland; 2.2 – inland other; Group II regions: 1. – border; 2. – midland.

Fourthly, there are indicators of resources concentration in the regions. They are used to assess the urban agglomeration effects and efficiency of using the territory of different types of regions.

## References

[1] Bell S., Pea A.C., Prem M. (2013). Imagine coastal sustainability. Ocean Coast. Manag.

[2] Kasanko M., Barredo J.I., Lavalle C., McCormick N., Demicheli L., Sagris V., Brezger A. (2006). Are European cities becoming dispersed? A comparative analysis of fifteen European urban areas. Landsc. Urban Plan..

[3] Leontidou L. (1990). The Mediterranean City in Transition: Social Change and Urban Development.

[4] Salvati L., Forino G. (2014). A ׳laboratory׳ of landscape degradation: social and economic implications for sustainable development in peri-urban areas. Int. J. Innov. Sustain. Dev..

[5] Sayas J.P. (2006). Urban sprawl in the periurban coastal zones of Athens. Greek Rev. Soc. Res..

[6] Serra P., Vera A., Tulla A.F. (2014). Spatial and socio-environmental dynamics of Catalan regional planning from a multivariate statistical analysis using 1980s and 2000s data. Eur. Plan. Stud..

[7] de Suárez Vivero J.L., Rodríguez Mateos J.C. (2005). Coastal crisis: the failure of coastal management in the Spanish Mediterranean region. Coast. Manag..

[8] Mikhaylov A.S., Mikhaylova A.A., Kuznetsova T.Yu (2018). Coastalization effect and spatial divergence: segregation of European regions. Ocean Coast. Manag..

